# TGF-beta Increases Permeability of 70 kDa Molecular Tracer from the Heart to Cells of the Osteoarthritic Guinea Pig Knee Joint

**DOI:** 10.3390/cells14191524

**Published:** 2025-09-29

**Authors:** Lucy Ngo, Melissa L. Knothe Tate

**Affiliations:** 1 MechBio Team, Graduate School of Biomedical Engineering, University of New South Wales, Kensington, NSW 2052, Australia; lucy.ngo@unsw.edu.au; 2 Blue Mountains World Interdisciplinary Innovation Institute, Blue Mountains National Park, Blue Mountains, NSW 2783, Australia

**Keywords:** osteoarthritis, cytokines, immune-circulatory-musculoskeletal systems, barrier function, tissue compartments, tight junctions, pathophysiology

## Abstract

Osteoarthritis involves complex interactions between articular joint tissues and the immune system, which is implicated in molecular trafficking via barrier-function modulating cytokines. The current study aims to test effects of an acute spike in TNF-α or TGF-β on vascular barrier function at multiple length scales, from the heart to tissue compartments of the knee, and cellular inhabitants of those respective compartments, in a spontaneous guinea pig model of osteoarthritis. First we quantified the intensity of a fluorescent-tagged 70 kDa tracer, similar in size to albumin, the most prevalent transporter protein in the blood, in tissue compartments of bone (periosteum, marrow space, compact bone, and epiphyseal bone) and cartilage (superficial cartilage, calcified cartilage, and the interface between, i.e., the epiphyseal line), as well as at sites of tendon attachment to bone (entheses). We then examined tracer presence and intensity in the respective pericellular and extracellular matrix zones of bone and cartilage. Acute exposure to TGF-β reduced barrier function (increased permeability) at nearest vascular interfaces in four of eight tissue compartments studied, compared to TNF-α where one of eight tissue compartments showed significant diminishment in barrier function. The increase in permeability associated with reduced barrier function was observed at both tissue compartment and cellular length scales. The observation of pericellular transport of the albumin-sized molecules to osteocytes contrasts with previous observations of barrier function in healthy, untreated animals and is indicative of increased molecular transport in pericellular regions of musculoskeletal tissues in cytokine-treated animals. Understanding age- and disease-related changes in molecular transport within musculoskeletal structures, such as the knee joint, is crucial for elucidating the etiology and pathogenesis of osteoarthritis.

## 1. Introduction

The cellular inhabitants of musculoskeletal tissues rely on patent molecular transport pathways [1,2] and intact functional transport barriers [3] between tissues to maintain viability in tissue habitats of intrinsically diverse structure and function. Osteoarthritis (OA), the most common musculoskeletal disorder, is characterized by chronic degeneration of the synovial joints, and is accompanied by pain and disability. While early studies of OA pathogenesis and progression focused on cartilage degradation, recent research has reconsidered OA as a multifactorial disease affecting all tissues comprising the joint [4,5]. Similarly, while OA was long-regarded as a non-inflammatory condition, contemporary paradigms recognize the presence of local inflammation in association with and as a major driver of OA progression [6,7,8]. Concomitant to local inflammation, age-related low-grade systemic inflammation is linked to OA and has been identified as playing a key role in OA’s pathogenesis [9]. OA patients exhibit elevated levels of serum inflammatory cytokines [10]. Two such cytokines, TNF-α and TGF-β, have been implicated as inflammatory mediators of OA and are thought to have a critical role in progression of OA [1,6,10,11,12].

Cytokines regulate immune cell function and differentiation, and their “dysregulation” is “associated with various human diseases” [13]. Cytokines TNF-α and TGF-β are involved in immune regulation and inflammatory responses and have been shown to exhibit both synergistic and antagonistic effects [14]. TNF-α has been shown to increase intestinal permeability by increasing intestinal epithelial tight junction permeability [15]. In contrast, TGF- β has been shown to reduce changes in tight junction proteins induced by TNF-α [14]. Compromise to intestinal barrier function via increased permeability has been identified as “an important pathogenic factor contributing to the development of intestinal inflammation” [15]. The role of molecular permeability and inflammation in osteoarthritis is poorly understood.

The interfaces between the cardiovascular and musculoskeletal system are vital to the maintenance of cellular and joint tissue health, and serve as the gateway for nutrition, waste removal and the delivery of regulatory factors to cells within the musculoskeletal system [2]. These vascular and extravascular pathways allow molecular transport to and between tissues and their cellular inhabitants [2,16,17,18,19,20,21]. It has been shown that the tissues of the joint exhibit the functional barrier property of molecular size and charge selectivity, with changes to permeability with age and/or disease state [1,16,17,18,19,20,21,22,23,24,25]. As noted above, the dynamics of blood-to-tissue barrier function have been shown to be modulated by TNF-α and TGF-β across numerous tissues with underpinning mechanisms related to alteration in tight junction structure and function, and thereby permeability [15,26,27,28,29].

**Figure 1:**
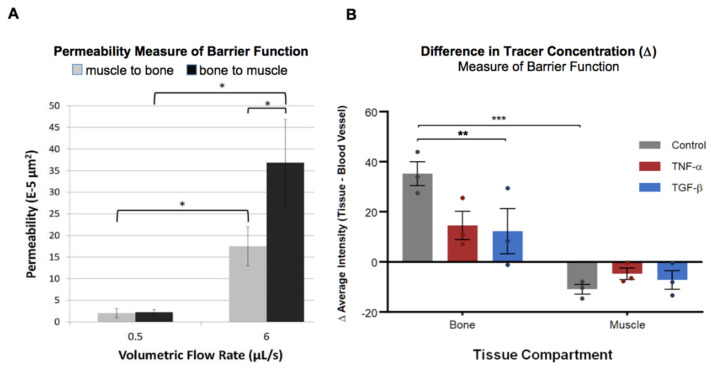
Hydraulic resistance and molecular tracer permeability in bone, muscle, and periosteum (tissue sheath, between bone and muscle, and bounding all non-cartilaginous outer surfaces of bone). (**A**). Flow resistance studies of ovine femur periosteum indicate that hydraulic permeability is direction- and flow rate-dependent; the periosteum is more than twice as permeable in the bone-to-muscle direction compared to the opposite direction. Significance is indicated by *: *p* < 0.05. *Figure reproduced with permission* [3]. (**B**). Episcopic cryoimaging measures fluorescent tracer intensity (a measure of concentration [24]) and shows barrier function (i.e., the difference in concentration of the tracer, cf. Figure 2) at vascular interfaces of bone and muscle in guinea pig knee joints. In untreated control tissues, barrier function at vascular interfaces is significantly higher in bone than in muscle (**: *p* < 0.001). In bone, treatment with either cytokine TNF-α or TGF-β reduces barrier function, with a significant reduction in the TGF-β group (**: *p* < 0.05, *** *p* < 0.001). *Figure reproduced with permission* [1].

While many studies have focused on molecular transport in cartilage and at the osteo-chondral junction [30,31,32,33,34,35], few have examined the multitude of other tissue interfaces that exist within the joint, or their intersection with the cardiovascular system, or how they may be modulated by immune events. 2014 studies from Evans et al. demonstrated that ovine periosteum, the sheath bounding all non-cartilaginous outer surfaces of bone, expresses tight junction protein ZO-1 [3,29]. Furthermore, flow resistance studies indicate a direction- and flow rate dependence of ovine femur periosteum permeability, where the periosteum is more than twice as permeable in the bone-to-muscle compared to the muscle-to-bone direction (Figure 1A) [29]. A 2023 study from our group showed that a spike in the inflammatory cytokines TNF-α or TGF-β, delivered via the heart in mature Dunkin–Hartley guinea pigs with osteoarthritis, increases permeability at the interface of the vascular and bone tissue compartments compared to the untreated baseline control animals (Figure 1B) [1].

The study compared fluorescence intensity of a fluorescent-tagged 70 kDa dextran, chosen as an inert surrogate for albumin, the 67 kDa, the most prevalent transporter protein in the blood. The intensity (directly proportional to concentration [36]) of the fluorescent-tagged dextran was measured using cryo-episcopic fluorescent imaging at near-single-cell resolution (10.2 × 10.2 μm in plane resolution) [1]. Permeability increases (i.e., reduction in barrier function) were significant for the pro-inflammatory cytokine TGF-β group. These observations of changes to barrier function provided the impetus for the current study comparing effects of a spike in TNF-α versus TGF-β with respect to the multi-scale transport of the 70 kDa fluorescent tracer from the heart to cells residing within specific bone and cartilage compartments within the knee joint of osteoarthritic guinea pig knees.

**Figure 2:**
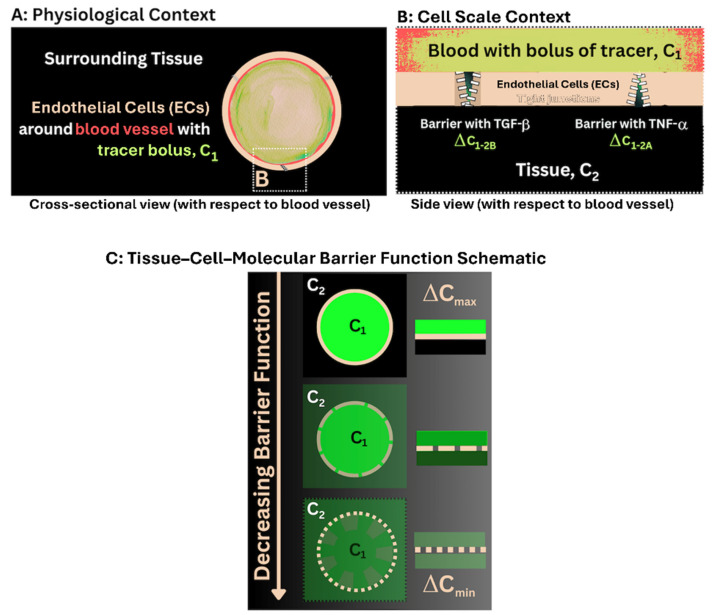
Barrier Function Schematic depicting a multi-length scale assessment of barrier function between tissue compartments. (**A**) Vascular-tissue interfaces exhibit a barrier function attributable to *i.a.* tight junctions between endothelial cells lining blood vessels, thereby controlling the passage of molecules across the interface. When a bolus of the fluorescent tracer is delivered via the blood vessel, the difference in concentration (Δ) across the interface (C_1_–C_2_), i.e., the intravascular concentration minus the tissue concentration (**B**), provides a quantitative measure of barrier function at that time point. (**B**,**C**) In the current study we hypothesize that acute increases in OA-associated inflammatory cytokines differentially decrease the barrier function of vascular tissue interfaces within the osteoarthritic knee joint.

Our overarching hypothesis was that acute increases in OA-associated inflammatory cytokines differentially decrease the barrier function of vascular tissue interfaces within the osteoarthritic joint (Figure 2). Our approach was to compare the immediate effect of a spike in inflammatory cytokines on barrier function at vascular tissue interfaces within the tissue compartments, and in the pericellular and extracellular zones of bone and cartilage, of the osteoarthritic guinea pig knee joint. For this study we used high-resolution imaging modalities (confocal and electron microscopy) after injection of a fluorescent-tagged fixable dextran, and its fixation and embedding in plastic resin.

## 2. Materials and Methods

### 2.1. Experimental Methods

All study methods were conducted in accordance with the ARRIVE guidelines and regulations. The study was approved by the Institutional Animal Care and Use Committee (IACUC) at Case Western University (Protocol #2012-0100, first approved in 2012 with addendum to the protocol for the current study (7 February 2014) and final protocol assessment on 18 January 2023). The study was approved as a series of experiments in which new independent variables were added to probe effects of age and disease state, molecular tracer size, co-delivery with cytokines TGF-β (R&D Systems, Minneapolis, MN, USA) and TNF-α (R&D Systems, Minneapolis, USA) [1,4,22], and comparative effects of the two cytokines on barrier function at tissue interfaces (current study) in the knee joint of Dunkin–Hartley guinea pigs. The Dunkin–Hartley strain develops osteoarthritis with increasing age and thus serves as an animal model for the human condition. Sample size (*n* = 3 per group) was calculated based on a power calculation and previous murine model data [37,38].

Six male Dunkin–Hartley guinea pigs, 11–13 months of age (Charles River, Wilmington MA, USA) were anesthetized via isoflurane and subsequently slowly injected, via the heart, with a single bolus of 70 kDa Texas-red tagged dextrans (Life Technologies, Carlsbad, CA, USA) and either TNF-α or TGF-β. The fluorescent tracer was chosen as a biologically inert surrogate for 67 kDa albumin, which has physiological significance as the most prevalent transporter protein in the blood and is at the molecular size limit for passage, e.g., through tissue interfaces of the knee joint. The tracer was prepared and delivered at a concentration of 1 µg/g body weight according to previously described protocols [1,2,22,24,25,36,39,40]. TNF-α or TGF-β were dissolved in 0.9% sterile saline solution resulting in dosages of 0.66 ng/g (TNF-α) and 3.33 (TGF-β) ng/g body weight. After five minutes of circulation time post-injection (during which guinea pigs remained anesthetized), animals were euthanized. The circulation time period was chosen to match previous studies in guinea pigs and rodents [1,2,22,24,25,36,39,40]. This timing also was chosen to mimic short cytokine spikes in contrast to continuously raised cytokine levels typical for chronic inflammatory conditions like osteoarthritis. Immediately after 5 min of tracer circulation, the knee joints were resected, with care taken to maintain the integrity of the joint including periarticular musculature. Hindlimbs were prepared en bloc for imaging as previously described [41].

### 2.2. Imaging

#### 2.2.1. Confocal Laser Scanning Microscopy

Confocal laser scanning microscopy was performed on the specimens using a Zeiss LSM 880 (ZEISS, Oberkochen, Germany), equipped with a 20 × 0.8 Plan-Apochromat dry objective and 63 × 1.4 Plan-Apochromat oil objective, at excitation and emission wavelengths chosen to observe tissue autofluorescence and the Texas-red fluorophore. The resulting 5000+ images per block face were stitched and rendered, creating navigable maps, using the Google Maps API (Google LLC, Mountain View, CA, USA) per methods previously developed for the visualization and analysis of electron microscopy of human-sized joints [40,41,42,43,44]. For quantification of tracer intensities, the images were scaled down by a factor of 0.25 using Arivis Vision4D (Arivis AG, Munich, Germany) and exported to TIF format. Tissue compartments in the region distal to and including the epiphyseal plate—superficial cartilage, calcified cartilage, bone, marrow, the epiphyseal plate, epiphyseal bone, periosteum, bone microvasculature, and tendon attachment sites (entheses)—were manually segmented in Fiji version 1.54 (Fiji Is Just ImageJ, Bethesda, MD, USA). Heatmaps of tracer density (Figure 7) were computed using MATLAB R2020a (MathWorks Inc., Natick, MA, USA).

#### 2.2.2. Backscattered Electron Scanning Electron Microscopy

To further decipher the interactions of the tracer with musculoskeletal tissue and cells, the specimens were stained with iodine and potassium iodine to enhance the contrast of cellular and soft tissue components according to published protocols [23,40,45]. The specimens then underwent backscattered electron scanning electron microscopy performed on a FEI NanoSEM 450 (Thermo Fisher Scientific, Waltham, MA, USA).

### 2.3. Statistical Analysis

Two-way ANOVA statistical analysis and Sidak’s multiple comparisons post hoc analysis was performed for comparisons of the tracer across groups and between multiple tissue compartments. *p* < 0.05 was considered significant; means and standard errors were reported. All statistical analysis was performed using GraphPad Prism 8 (GraphPad Software, San Diego, CA, USA).

### 2.4. Sample Size Calculations

For the serial experiment study design, sample size was computed using a power analysis in which the expected difference between controls and groups is 50% and variability around means is 15%. Using an alpha level of 0.05, these differences could be detected with a power of 0.90 by having a minimum sample size of three. The power calculations were validated post hoc using previous data from this series of guinea pig experiments and by molecular transport studies using different imaging modalities for outcome measurements in a murine model [1,2,4,22,24,25,36]. For the current comparative study, we used the same sample size for each group. In this way, we maximized study power and insights while minimizing animal numbers, an essential aspect of ethical considerations in accordance with the “first R” of the 3Rs of ethical animal experimentation, i.e., reduction in animal numbers to a minimum required for reliable data [37,38].

### 2.5. Specimen Fixation and Preparation for Imaging

Following immersion fixation in 4% paraformaldehyde at 4 °C, the specimens were dehydrated via increasing ethanol sequence for polymethylmethacrylate (PMMA) embedding. Once embedded, the specimens were sectioned to expose the tibial-femoral (knee) joint. The joint surface was precision milled in a CNC-lathe to ensure planarity of the specimen and polished to a mirror-like finish [23,40].

## 3. Results

**Figure 3:**
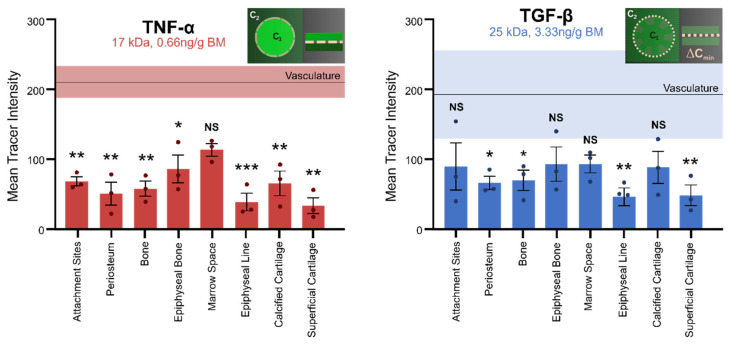
Effect of an acute increase in inflammatory cytokines TNF-α or TGF-β on barrier function. Barrier function is assessed quantitatively as the difference (Δ) in intensity (concentration [29]) of the 70 kDa tracer intensity between the vascular and specific tissue compartments within the joint. A significant difference in concentration between the vascular and a specific tissue compartment indicates intact barrier function; conversely, no significant difference in concentration indicates a lack of barrier function between the respective compartments. Significant (*, **, ***) differences in concentrations between tissues of the knee and the vascular compartment are observed for each tissue in the TNF-α-exposed group, except for the marrow space, indicative of barrier function in 7 of 8 tissues studied. Significant differences in tracer concentrations are only observed in the periosteum, (compact) bone, epiphyseal line, and superficial cartilage of the TGF-β exposed group, indicative of abrogation of barrier function in 4 of 8 tissues measured. The shaded horizontal bands indicate the ranges of mean tracer intensity +/− SEM for respective vascular compartments of the TNF-α and TGF-β groups. Significant differences were determined by two-way ANOVA and Tukey’s multiple comparisons post hoc analysis. Error bars indicate standard error (*n* = 3). Statistical significance indicated by * (*p* < 0.05), ** (*p* < 0.01), and *** (*p* < 0.001).

We tested the hypothesis that acute exposure to increased levels of TNF-α and TGF-β, delivered via the heart exert different effects on barrier function between tissue compartments of the osteoarthritic knee joint. Barrier function was assessed by quantifying intensity differences between vascular and different specific tissue compartments (Figure 3), noting that intensity serves as a surrogate for concentration [36]. Hence, differences could be captured in two ways: (i) difference between vascular and specific tissue compartments (x-axis, Figure 3) and (ii) number of different tissue compartments showing significant differences in concentration compared to the vascular compartment (denoted with * for significance, *p* < 0.05).

### 3.1. Immediate Cytokine-Modulated Transport Within the Joint

The functional barrier between the vasculature and the respective tissue compartments of the knee joint was measured as the difference in the mean concentration between the two. In the group exposed to an acute increase in the pro-inflammatory cytokine TNF-α (Figure 3, left panel), a significant difference in mean tracer intensity was observed between the vasculature and all tissue compartments except the marrow space, indicative of a functional barrier between the vasculature and all respective tissue compartments except the marrow space (i.e., in seven of eight tissue compartments studied). After an acute increase in the anti-inflammatory cytokine TGF-β (Figure 3, right panel), barrier function was significantly diminished and showed fewer significant differences in mean tracer intensity between the vasculature and respective tissue compartments (i.e., in four of eight tissue compartments studied, Figure 3), indicative of higher permeability.

No significant differences in tracer concentration could be observed between specific tissue compartments, including (bone) attachment sites, periosteum, (compact) bone, epiphyseal bone, marrow space, epiphyseal line, calcified cartilage, and superficialcartilage—neither within nor between the respective compartments of the TNF-α and/or TGF-β groups (Figure 3).

The vasculature of the TNF-α group exhibited a mean intensity of 210.5 ± 22.6, ranging from 167.4 to 243.8. The vasculature of the TGF-β group showed a slightly lower mean intensity of 192.5 ± 63.5. The range of intensities for vascular compartments in TGF-β-exposed animals is circa 2.8 times that of the respective TNF-α-exposed vascular compartments, indicative of loss in barrier function. Tissue compartments are organized (left to right) roughly from outside–in (in a cross sectional context) and distal to proximal (to a longitudinal plane) to the articular surface, i.e., tendon insertions to bone (attachment sites), periosteum covering the outer surface of bone (periosteum), compact bone (bone), epiphyseal bone, bone marrow/medullary space (marrow space), epiphyseal line, calcified cartilage, and superficial cartilage.

### 3.2. Molecular Transport from the Heart to the Cellular Inhabitants of the Musculoskeletal System

Correlation of relevant anatomical and physiological features, in high-resolution, stitched (tiled) confocal microscopy images (40× and 63×), and backscattered electron microscopy images (100–1000×) was used to visualize structural and functional properties within and at interfaces between tissue compartments. Following vascular pathways from the heart to the respective tissue compartments of the knee, highest concentrations of the 70 kDa molecular tracer were observed as intense red fluorescence in the popliteal artery and vein, and to a lesser degree in the marrow space (Figure 4A). The marrow space showed regions of high tracer concentration; the tracer appeared granular and clustered around the dark adipose cells (Figure 4B,D). Muscle tissues exhibited diffuse, low levels of the tracer, with bone exhibiting even lower levels of the tracer compared to muscle. The synovial fluid was devoid of the tracer (Figure 4A).

**Figure 4:**
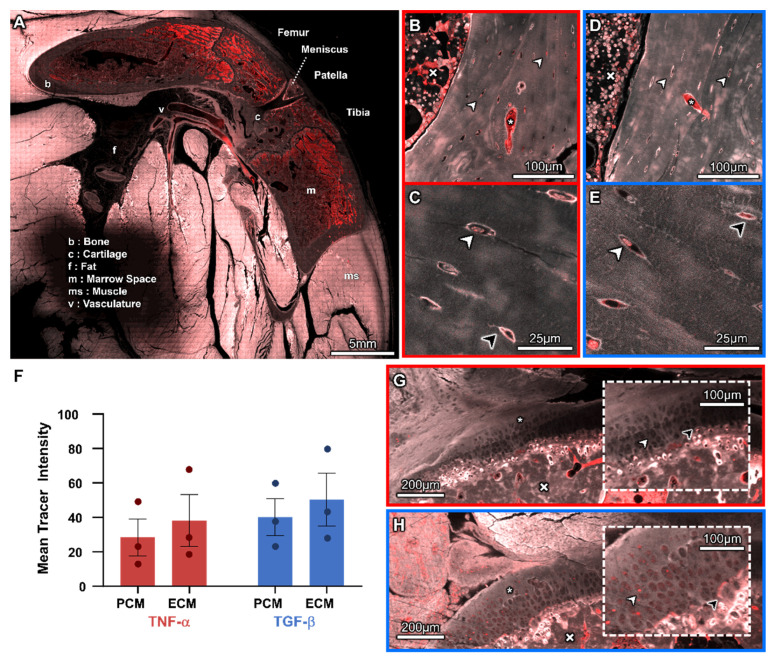
Molecular transport from the heart to the cellular inhabitants of the musculoskeletal system, accounting for presence of the tracer (70 kDa) in PCM (pericellular matrix) and ECM (extracellular matrix) regions of bone and cartilage. (**A**) Overview of a sagittal cross section of the knee, with the patella out of the sectional plane. Gross molecular separation of the tracer (red) in autofluorescent musculoskeletal tissues (white), is visualized using circa 5168 confocal microscopy fields of view. At higher magnification, some regions show the tracer within the vasculature and osteocyte lacunae in (**B**,**C**) TNF-α and (**D**,**E**) TGF-β groups; crosses (x) denote marrow and asterisks (*) indicate vasculature. Light and dark arrowheads point to the tracer within intracellular and/or pericellular regions of osteocytes, respectively. (**F**) Comparison of mean tracer intensity in the PCM and ECM of cartilage in the TNF-α and TGF-β groups. No significant differences were noted between cytokine groups or regions of interest (two-way ANOVA and Tukey’s multiple comparisons post hoc analysis). (**G**,**H**) Tracer distribution in the superficialcartilage (indicated by asterisks, *) versus subchondral bone (indicated by crosses, x), where light and dark arrowheads (insets) point to chondrons in the calcified joint cartilage layer and the tidemark, respectively. Animals from the TNF-α group (**F**,**G**) exhibited lower levels of the tracer in the ECM, and even less of the tracer in the PCM compared to the TGF-β group (**F**,**H**). The TGF-β group exhibited similar patterns of the tracer in the cartilage than the TNF-α group but was distinct in exhibiting elevated tracer permeation at the tidemark and within the chondron (not observed in the TNF-α group (**G**,**H**).

Within the specific tissue compartments of bone and cartilage, differences in pericellular and extracellular transport were observed in association with acute exposure to inflammatory cytokines. Within bone tissue of the TNF-α and the TGF-β groups, elevated levels of the tracer were observed within osteocyte lacunae, within the pericellular matrix (PCM) and surrounding canaliculi. Within the osteocyte itself, the tracer was observed to be concentrated in the perinuclear area where the exosomes are located (Figure 4B–E), indicative of endocytic transport.

In cartilage tissue of both the TNF-α and TGF-β groups, the calcified cartilage exhibited higher intensities of tracer than the underlying bone and capping superficial cartilage, increasing with proximity to the articular surface; higher concentrations of the tracer were present at the boundaries of hypertrophic chondrocytes. Both the TNF-α and TGF-β groups exhibited small amounts of the diffuse tracer in the superficial cartilage matrix with higher levels of the tracer at the articular joint surface. With respect to the cartilage, we observed qualitatively less tracer in the pericellular matrix (PCM), which appeared darker than the surrounding extracellular matrix (ECM). However, no statistical significance was seen in mean tracer intensities between PCM and ECM in cartilage tissue or between the TNF-α and TGF-β groups (Figure 4F).

Qualitative observations of the TNF-α samples revealed little to no tracer in the PCM in all regions of the cartilage (Figure 4G). In contrast, animals in the TGF-β group displayed raised levels of the tracer within the chondron (PCM) in comparison to the surrounding ECM. Accumulation of the tracer was also observed at the tidemark in the TGF-β group, which were not present in the TNF-α group (Figure 4H).

**Figure 5:**
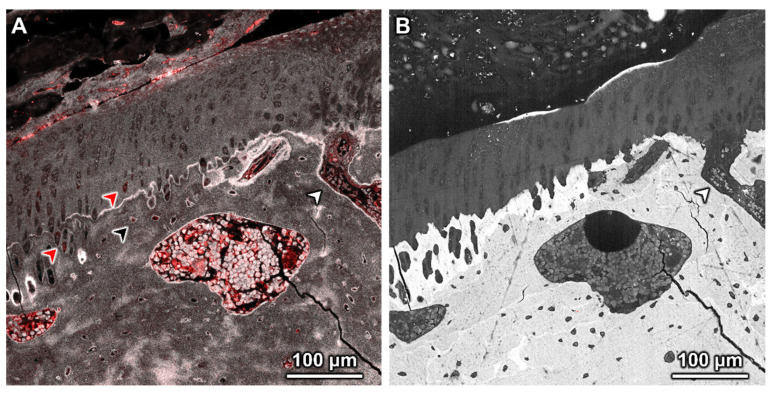
Multimodal imaging of the osteochondral junction—cartilage, subchondral bone, and underlying marrow spaces in a sample from the TGF-β group. The white arrow points to the marrow space that extends past the subchondral bone and calcified cartilage into the superficial and mid to deep zones of non-calcified cartilage. (**A**) Confocal microscopy with 70 kDa dextran appearing red and tissue autofluorescence in white (gray). Red and black arrows indicate chondrocytes and osteocytes, respectively, with greater levels of tracer compared to other cells in the region of interest. (**B**) BS-SEM image of iodine stained, and carbon-coated specimen show calcified and non-calcified tissue regions. Calcified cartilage appears brighter than surrounding bone. Red blood cells (indicated by white arrowhead) appear within a vessel in a region of marrow that extends into the calcified cartilage. The crack in the lower right corner is an artifact of sample preparation.

Based on high-resolution confocal microscopy images, the cartilage surfaces of all animals appeared largely intact with some areas exhibiting small amounts of superficial fibrillation; no duplication of the tidemark was detected. The calcified cartilage exhibits slightly increased levels of autofluorescence compared to surrounding cartilage and subchondral bone. We observed increased levels of the tracer sporadically in osteocytes within the subchondral bone; in addition, the TGF-β group exhibited elevated levels of the tracer in some chondrocytes. In some animals, the marrow space appeared to extend through the subchondral bone, and the calcified cartilage appeared to merge into the cartilage, with the tidemark less apparent at this junction (Figure 5A).

**Figure 6:**
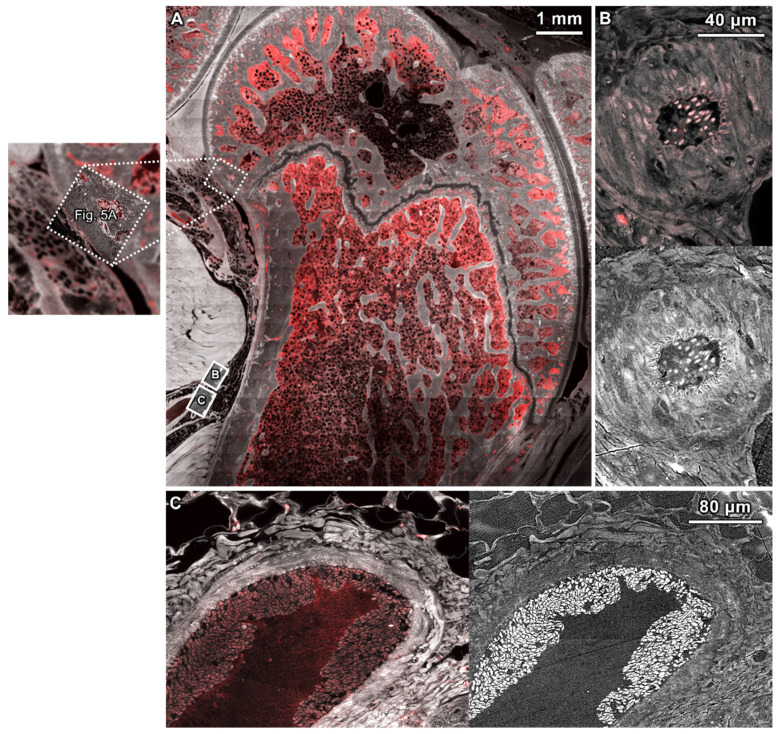
Imaging at the interface of the cardiovascular and musculoskeletal systems demonstrates the power of multiscale, multimodal, and correlative imaging, e.g., of the guinea pig distal femur exposed to an acute increase in TGF-β. An overview image of the guinea pig distal femur (**A**) comprises tiles of confocal microscopy images with overlaid regions of interest acquired with BSE-SEM. The correlated confocal and electron microscopy region of interest from Figure 5A is depicted by the dotted white square (**left inset**). Confocal ((**B**,**top**) and (**C**,**left**)) and BSE-SEM ((**B**,**bottom**), and (**C**,**right**)) images depict the popliteal vasculature ((**B**)—popliteal artery, (**C**)—popliteal vein). Confocal images help in visualizing relationships between the fluid elements of the vascular system (red, 70 kDa tracer) where albumin-sized (67 kDa) molecules would permeate, as well as the musculoskeletal tissue matrices (PCM as well as the ECM, see Figure 4). Electron microscopy highlights cellular inhabitants in the vascular and extravascular tissues and structure–function relationships of the cardiovascular system. Correlative imaging allows concomitant visualization of these elements for any given field of view of interest, from nano- to meso-length scales.

Subsequent correlative SEM images of the same region of interest showed deterioration of the cartilage surface (Figure 5B). These areas were observed in confocal images (Figure 5A). In the SEM images, some areas appear white, indicative of a change in cartilage composition (increased mineralization) compared to adjoining surface regions (Figure 5B). Referencing the overview image of this region of interest (Figure 5A, dotted square Figure 6), the area of cartilage degeneration is at the metaphysis, near the epiphyseal plate. The boundaries of calcified cartilage are more apparent in SEM images; however, the tidemark is not visible. The images reveal small vessels containing red blood cells within the open connection between the marrow space and calcified cartilage (Figure 5B); these were not discernable in the confocal images of the same region of interest (Figure 5A).

**Figure 7:**
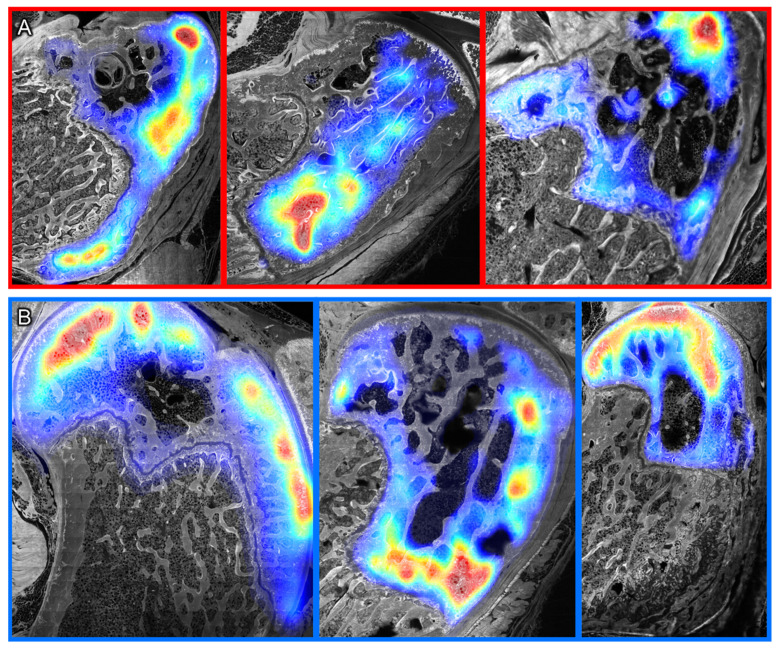
Tracer heatmaps of the distal femur up to the epiphyseal plate indicate tracer separation generally aligning with musculoskeletal tissue compartments in (**A**) TNF-α and (**B**) TGF-β groups. Warm colors (yellow => orange => red, increasing “warmth” or concentration) indicate increasing, higher concentrations than cool colors (green => turquoise => blue, decreasing “warmth” or concentration, respectively increasing “cool”).

Finally, to better visualize the spatial distribution of the fluorescent tracer, a heat map depiction of tracer intensity, with warm colors marking areas of high tracer concentration and cool colors showing low tracer concentration, was overlaid onto the stitched confocal images of the joint surface in the sagittal plane (serial sections). The spatial heterogeneity of tracer distribution was grossly observable; the spatial separation of “hot spots” and “cool spots” generally aligns within the boundaries of tissue compartments, from the superficial cartilage of the articular joint surface to the epiphyseal line of the distal knee (Figure 7).

## 4. Discussion

In a spontaneous model of osteoarthritis, we compared the barrier function at vascular—tissue interfaces of the joint after five minutes of exposure to a sudden increase in TGF-β versus TNF-α. Exposure to a spike in TGF-β is associated with diminished barrier function (molecular permeability) at interfaces between the vascular system and four of eight tissue compartments making up the osteoarthritic knee joint. In contrast, exposure to a spike in TNF-α is associated with diminished barrier function in only one of eight respective tissue compartments. Based on the results of this multimodal, multiscale, and correlative imaging study, TGF-β exposure may diminish barrier function (increase permeability) more than TNF-α exposure, although further studies of dose-related effects and temporal pharmacokinetics are warranted. These data corroborate our previous study using lower-resolution episcopic cryoimaging of bone and muscle [1]; furthermore, they implicate the capacity of acute events, such as trauma and cytokine storms induced by viral disease, to significantly impair barrier function between tissues and thereby molecular trafficking, even in osteoarthritic synovial joints which would be exposed to chronic inflammation. In addition, the study implicates cytokine-induced changes to pericellular and extracellular transport of the large molecular tracer (70 kDa) that mimics transport of albumin (67 kDa), the main carrier protein in the blood plasma. Exposure to both cytokines appeared to increase transport of the large-molecular-weight tracer to the pericellular region of chondrocytes (the chondron), which may offer a means to open transport pathways for concurrent delivery of pharmaceutical agents that improve chondrocyte viability as well as matrix regeneration by chondrocytes to enable cartilage repair in OA patients [46]. As a whole, the study provides new insights into pathways of transport between the cardiovascular system and the cellular inhabitants of the musculoskeletal system. From a methodological and mechanistic perspective, the study underscores the power of high-resolution correlative imaging to enable an integrated understanding of complex biosystems and their cellular inhabitants.

A unique advantage of the multimodal correlative imaging approach of the current study is that the loss in structure and function associated with OA can be linked seamlessly across organ-to-subcellular-length scales. This facilitated the visualization and quantification of the tracer from the heart to the cells populating the respective tissues of the synovial joint (Figure 4, Figure 5, Figure 6 and Figure 7); akin to tracking truck traffic in road transport networks, future imaging modalities may enable real-time assessment of traffic dynamics which would be invaluable for understanding mechanisms of pathophysiology, novel drug delivery strategies, and new preventative health measures [23,41,42,43,47]. The current study highlights the near instantaneous nature of the circulation to the joint, with the dextran tracer perfusing all musculoskeletal tissues within the five-minute circulation period of the study. The integrated, correlative, and multimodal imaging approach also enabled connection of articular surface degeneration at smallest length scales to mesoscopic biomechanical considerations of joint structure and function, where compositional changes in the surface observed with electron microscopy could be tied to the metaphysis of the joint, e.g., where stress concentrations would occur during physiological loading (Figure 4, Figure 5A and Figure 6). All knee joints studied showed cartilage surface fibrillation, and marrow space openings as well as vascular invasion into the calcified cartilage, with no tidemark duplication at the osteochondral interface; these all represent morphological features characteristic of early to moderate and, in some cases, severe osteoarthritis [4,22,32,46].

A limitation and strength of the study is that the Dunkin–Hartley guinea pigs spontaneously develop osteoarthritis with age, which precludes study of age-matched controls without OA yet provides a relevant model for the human condition, where onset of OA correlates with age [22,48,49]. Use of younger animals prior to onset of OA would not provide an appropriate independent control, since our previous studies using the same animal model, including a younger cohort, demonstrate an age as well as a disease-state dependence on distribution and intensity of fluorescence tracers [22]. Inclusion of non-arthritic, age-matched controls would have been ideal (as a measure of healthy baseline barrier function) but not possible using this animal model of age-related OA. Given that the baseline levels of TGF-β and TNF-α are affected by the presence of OA itself, our experimental design used a comparison between two groups exposed to acute spikes in TGF-β and TNF-α. While this allowed us to reduce animal numbers to a minimum required for reliable data [37,38], an additional group not exposed to an acute spike in cytokines should be included in future, allowing for mechanistic studies to elucidate both baseline barrier function in OA knees exposed to chronically ”skewed” plasma cytokine levels, as well as effects of a spike in pro- and anti-inflammatory cytokines on barrier function. To further elucidate mechanisms underpinning the cytokines’ effects on barrier function, it would also be beneficial to include high-resolution imaging of tight junctions at tissue interfaces in future studies.

Another recent study from our group, implementing episcopic cryoimaging, showed that exposure to an acute spike of inflammatory cytokine TGF-β significantly reduced vascular barrier function in bone and muscle compared to age-matched controls [1]. This observation provided the impetus for the current study, where plastic embedding and multimodal, higher resolution imaging modalities allowed for quantitative assessment of barrier function in different tissue compartments of the knee joint. For that reason, the study design compared tracer transport in two age-matched cohorts of the same species with similar stages of OA, where one group was administered a pro-inflammatory cytokine TNF-α and the other was administered an anti-inflammatory cytokine TGF-β.

In published studies of, e.g., the blood–brain barrier and intestinal mucosa, TNF-α and TGF-β have been shown to exert different [50,51,52] and/or opposite effects [52] on barrier function. At the onset of the current study (and to our knowledge, to date), no similar organ-to-subcell scale-work had been carried out at vascular tissue interfaces of the osteoarthritic knee joint; hence, we aimed to assess changes in barrier function due to an acute spike in either cytokine or in a spontaneous OA animal model ([1,22,48,49], aged Dunkin–Hartley guinea pigs). In addition, no reference norms were (or are) available for baseline plasma concentrations of TNF-α or TGF-β in healthy or osteoarthritic guinea pigs; thus, the experiment was designed to achieve an acute spike in cytokines (orders of magnitude over baseline levels) in osteoarthritic animals. To validate that exogenous delivery of the cytokines achieved an acute spike in the blood in the current study, we compared the dosages used with comparable cytokine levels in aged human subjects in different physiological contexts.

Firstly, we validated the relative dosages of TNF-α and TGF-β delivery. Recent studies show a circa four orders of magnitude higher concentration of TGF-β (3300.14 and 2897.37 pg/mL) compared to TNF-α (0.27 and 0.39 pg/mL) in blood plasma of, respectively, young (18–35 years) and aged (50 years and older) adult human subjects [53]. To place the current study in context, and taking a mean value of 3.94 mL plasma per 100 g body weight of a guinea pig [54], the delivered dosages of 0.66 ng/g (TNF-α) and 3.33 (TGF-β) ng/g body weight correspond approximately to 16,750 pg/mL and 84,520 pg/mL plasma, respectively, i.e., amounting to spikes of circa 43,000× and 29× baseline plasma values for TNF-α and TGF-β in aged human subjects. While not directly comparable, neither with respect to guinea pig vs. human nor with respect to the two cytokines studied, the respective spikes represent one (TGF-β) to four (TNF-α) orders of magnitude increases over baseline values in plasma of aged human subjects, confirming that an exogenous spike above baseline levels was delivered. The guinea pigs in the current study correspond to the human condition, with respect to age and OA status [1,22].

Secondly, to put the exogenous cytokine spikes in a physiological context, we turned to the recent literature on cytokine spikes in human patients hospitalized with COVID-19. An equivalent spike in TNF-α has been reported as 38× (mean) to as high as 260× above baseline for critically ill human COVID-19 patients with pneumonia [55]; in contrast, the equivalent spike in TGF-β for a different cohort of severely ill COVID-19 patients with raised platelet counts was 2× (mean) to as high as 3× above baseline [56]. Hence, in context of its presentation in human patients, the spike in cytokines administered in the current study was well above the increases observed in human plasma in response to a so-called “cytokine storm” [55,56], relative both to baseline TNF-α as well as TGF-β plasma levels.

Thirdly, the study design emulates those carried out to probe the blood–brain barrier and other tissue barrier functions in human patients and animal models [26,27,28,29,45,46,47]. For example, an increase in vascular albumin permeability was reported with exogenously administered TNF-α in in vivo mouse model studies of lung, liver, kidney, brain, sciatic nerve, retina, duodenum, jejunum, cecum, thoracic aorta, skin and diaphragm [57]. In contrast to the current acute study observing changes 5 mins after TNF-α delivery, significant increases were not observed in the previously published mouse study until 12–18 h after delivery of TNF-α. Furthermore, the published mouse study administered TNF-α at 20 ng/g [32], a dose greater than thirty-fold that of the current study. In addition to underscoring potential effects of not only dose-related but also temporal pharmacokinetics, these differences may also reflect intrinsic differences between the healthy murine model, spontaneous age-related OA in the guinea pig, and healthy or OA-affected human subjects.

Taken in these diverse physiological contexts, the results of the current study challenge a prevailing theory that TGF-β preserves epithelial barrier function (decreasing permeability) and TNF-α disrupts barrier function (increasing permeability) [57], giving impetus for further interdisciplinary approaches to understand spatiotemporal pharmacokinetics from organ-to-cellular-length scales. A limitation of the current study is that it was not possible to carry out a dose–response and/or spatiotemporal analysis of each cytokine studied due to resource constraints, both with regard to the animal models, access to chemical agents (cytokines), as well as intrinsic limitations of state-of-the-art imaging modalities (e.g., live imaging not yet possible). The current study, however, provides new insights to guide future investigations. Published reports of serum cytokine levels in healthy and diseased as well as young and aged human cohorts [1,3,13,14,15] are also increasing and will provide new foundations for future studies.

We observed compartmentalization of molecular transport within the joint similar to that reported in previous studies, and the significant changes in barrier function with exposure to TGF-β confirm those reported in previous cryoimaging studies [1,22]. These were observed irrespective of intrinsic differences in the technologies used for fluorescent imaging of episcopic specimens and laser scanning confocal microscopy (LSCM) imaging. LSCM, in general, allows for more precise and powerful excitation of fluorophores and capture of emitted light in targeted wavelengths. Further, differences between cryo- and chemically fixed tissues are expected to be minimal, since the cryofixed specimens are fixed and frozen using liquid nitrogen and OCT compared to the chemical fixation of lysine-fixable dextrans for multimodal imaging. Cryofixation occurs at such low temperatures that any potential diffusive processes are expected to be slowed, and chemical fixation of the “fixable dextrans” containing a primary amine chemically fixes the dextrans in place [58,59,60].

Previous experiments have demonstrated the molecular sieving properties of bone where diffusivity decreases with increasing molecular size; at 70 kDa, or circa 7 nm Stokes diameter, molecules are no longer able to pass through the bone lacunocanalicular system (LCS) [2,16,24,61,62]. Molecules of molecular weight in the range of TNF-α (17 kDa) and TGF-β (25 kDa) have been reported to permeate the lacunocanalicular system and may modulate barrier properties within the LCS. Here, we observed the 70 kDa molecule in the LCS which contradicts previously reports that molecules of this size are excluded from the LCS, even with the aid of mechanical stimuli and associated convective transport [2,24,36]. The circulation time of the tracer in the current study was chosen to match that of previous studies tracing transport from the vascular system to the osteocytes in healthy and acute fracture rodent models; sample sizes also matched those of previous studies [2,24,25,36,39]. Taken together, the current study suggests that the chronic OA disease state, combined with acute exposure to inflammatory cytokines TNF-α and TGF-β, increases LCS permeability and associated transport to osteocytes.

Similarly, the permeability of large molecules in cartilage has been shown to be largely dependent on cartilage composition, which itself depends on health or disease state [63]. It has been demonstrated that changes brought on by OA, i.e., cartilage degradation, also alter tissue structure and composition [64]. In the current study, we observed a small amount of the tracer within the articular cartilage, with elevated levels of 70 kDa tracer observed inwards toward the articular surface. This suggests that a greater permeability to macromolecules in the cartilage in the middle and deep zones is accompanied by decreased retention in the tissue. This is consistent with findings that different sized antibodies, e.g., 25 kDa, 50 kDa, 150 kDa, and 200 kDa in size, diffuse into the cartilage with greatest concentrations observed at the articular surface [65]. The diffusion characteristics of 70 kDa dextrans in cartilage PCM have been shown to change with disease state in a porcine model, where healthy animals exhibit significant differences in PCM and ECM diffusion coefficients, and OA animals do not [66]. This is also consistent with our findings that the differences in tracer levels in ECM and PCM were not significant in the guinea pigs which spontaneously develop OA with age. However, the accumulations in the chondron in TGF-β animals but not TNF-α animals suggest that TGF-β may differentially change permeability characteristics of articular cartilage, providing impetus for further studies.

**Figure 8:**
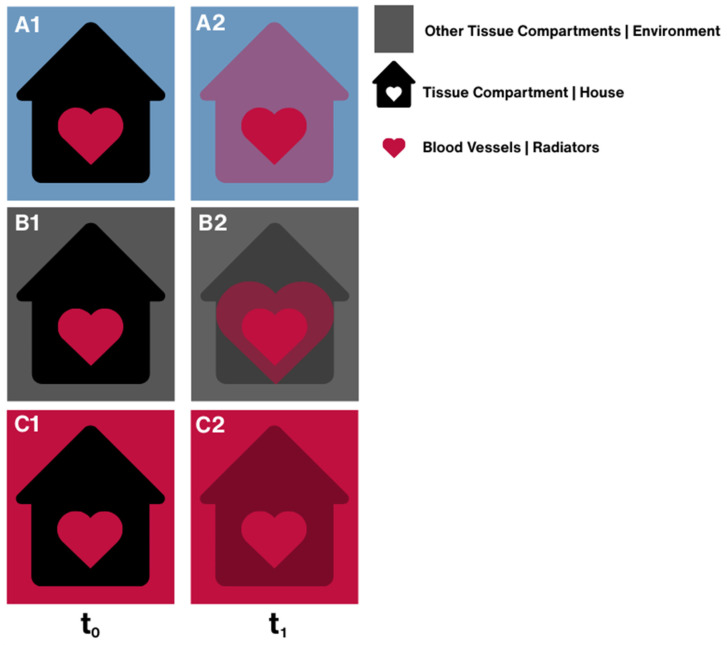
Heat transfer analogy for how barrier function impacts the transport of the fluorescent-tagged tracer from the heart to cells inhabiting tissue compartments. (**A1**) If the temperature outside is cold (low concentration of the tracer) and the house is cold, with time, heat from the radiator will warm the house (**A2**). Depending on the house insulation, this heat may further dissipate to the surrounding environment. (**B1**) If the house and its environment are of a similar temperature (similar tracer concentrations) but the house is colder (smaller concentration) than the radiator, then the radiator will dissipate heat (**B2**) and warm the house (increasing concentration). (**C1**) If the environment is warmer (higher concentration) than the house, the house will warm up (increase in concentration, depending on barrier function/house insulation) from outside–in, and if the radiators are warmer than the interior of the house, the house will warm up from the inside (**C2**).

A comparison of tissue compartments between and across the TNF-α and TGF-β groups did not demonstrate significant differences in bulk permeability of respective tissue compartments. However, the data did show that there was a significant modulation of transport between the bone microvasculature and other tissue compartments in the femur in the respective groups. It is helpful to use a heat transfer analogy (from a physics/mathematics perspective, molecular transport and heat transfer are analogous) to visualize how barrier function impacts transport of the fluorescent-tagged tracer from the heart to cells inhabiting tissue compartments (Figure 8). We depict respective tissue compartments as houses, where the barrier function of the tissue itself is analogous to house insulation at boundaries to the environment. The blood vessels within the tissue have their own barrier function, for which we make the analogy of a radiator, where the barrier function would be the efficacy of heat transfer from the radiator. Finally, the tissue compartment’s surrounding environment (e.g., other tissue compartments within the joint) is depicted as the environment of the house. In each case, the rate of heat transfer depends on both the difference in temperature (concentration) across boundaries as well as the functional efficiency (insulation or heat transmission capacity) of the boundary. In the current study, we hypothesize that TGF-beta reduces barrier function (increases permeability) of tight junctions, which are found in different epithelial tissues and with different degrees of “permeability” when one compares those between endothelial cells lining blood vessels and those in the lining, e.g., the cambium layer of the periosteum or the entheses. The fluorescence intensity measurements of the current study are indicative of concentrations within specific compartments (tissues|environment vs. house) including the vascular compartment (blood vessels|radiators). Furthermore, in the current study, the exogenous spike in TGF-beta is associated with an increased range of concentration in the blood vessels and with a decrease in barrier function (concentration or temperature difference) between more tissue compartments and the vasculature than exposure to a spike in TNF-alpha. This can be best schematically depicted by B2 (Figure 8), since differences in concentration between respective tissue compartments were not statistically significant.

Between and across the TNF-α and TGF-β groups, no significant differences in barrier function were observed between tissue compartments. However, the data showed a significant modulation of transport between the bone microvasculature and other tissue compartments in the femur, in the respective groups. Although these cytokines may not necessarily modify the tight junctions at tissue compartment boundaries (this should be tested mechanistically in future studies), they do alter the function of the microvasculature within the joint. TGF-β has been shown to be vital to endothelial barrier function and thus maintenance of microvasculature, with an increase in TGF-β being implicated with the breakdown of the functional tissue barrier at the blood-retinal boundary [67,68]. Similarly, TNF-α has been shown to induce increased vascular permeability through endothelial dysfunction in the brain and lung [69,70]. There is mounting evidence of the association of vascular pathology and OA pathogenesis [32,66,71]. TNF-α and TGF-β were shown to have an immediate effect on vasculature, and it is hypothesized that cycles of intermittent increase in pro-inflammatory cytokines associated with age-related chronic inflammation may cause further deterioration of the functional barrier at vascular interfaces and may be a contributing factor to OA pathogenesis.

Visualization and quantification of macromolecular transport to and between tissue compartments of the joint, via the circulatory system and interstitial transport mechanisms, require imaging modalities and specimen preparation that allow for visualization across multiple length and time scales. The current study was not without limitation, as imaging in a single plane (comprising thousands of images) and at a single time point captures “a snapshot” of a dynamic process. An ideal imaging modality would allow for temporal assessment, in real time and across length scales. In vivo imaging, albeit currently available at much lower resolution, may provide an additional degree of temporal fidelity in the future, as imaging modalities and respective computational capacity increase in resolution and speed.

Furthermore, while the molecular weight of the dextran (70 kDa) is comparable to that of albumin (67 kDa), dextrans differ both in shape and molecular charge to globular proteins; these factors can affect the mobility of large molecules within tissues. The hydrodynamic radius is used as a measure for an equivalent sphere with an identical translational diffusion coefficient, e.g., the hydrodynamic radii of albumin (circa 3.5 nm) and dextran (circa 6.5 nm) [72], share similar orders of magnitude. However, published reports of size selectivity for 70 kDa dextran and albumin in vascular, lymphatic, and renal transport indicate that factors such as shape and charge merit further study [73]. Also, although we observed changes in barrier function between the two groups, in this present study it is not possible to separate the immediate effects of inflammatory cytokines and the chronic effects of exposure to inflammatory cytokines in osteoarthritic, age-matched subjects.

## 5. Conclusions

This study provides further evidence for inflammatory cytokines’ role in OA pathogenesis, revealing inflammation-induced changes to barrier properties between vascular and tissue compartments of joints exposed to TGF-β. These data provide further evidence relating to changes in bone–cartilage crosstalk to pathogenesis of osteoarthritis [32]. The study opens new avenues to harness effects of cytokine-driven transport to musculoskeletal tissues for drug delivery [23,74]. It also provides intriguing inspiration for engineering of next generation medical textiles with functional barrier properties [44]. The interplay between mechanical loading-induced active transport [2,16,17,18,19,24,36,39,41,65], mechanoadaptation [36,40,44,75,76,77], and systemic inflammation [1,21,25] remains unknown and provides impetus for further study.

## Data Availability

The datasets generated and/or analyzed during the current study are available in the MechBio data repository on the Blue Mountains World Interdisciplinary Institute website https://mechbio.org or https://blueinnovations.org, under the section “Data Repositories”. In addition, the datasets used and/or analyzed during the current study are available from the corresponding author on reasonable request.

## Abbreviations

The following abbreviations are used in this manuscript:

OA	Osteoarthritis
TNF-α	Tumor necrosis factor alpha
TGF-β	Transforming growth factor beta
ZO-1	Zonula occludens-1
ARRIVE	Animal research: reporting of in vivo experiments
IACUC	Institutional animal care and use committee
PMMA	Poly(methyl methacrylate)
CNC	Computer numerical control
CLSM	Confocal laser scanning microscopy
SEM	Scanning electron microscopy
BSE-SEM	Backscattered electron scanning electron microscopy
API	Application programming interface
PCM	Pericellular matrix
ECM	Extracellular matrix
